# Galectins in epithelial-mesenchymal transition: roles and mechanisms contributing to tissue repair, fibrosis and cancer metastasis

**DOI:** 10.1186/s40659-024-00490-5

**Published:** 2024-04-04

**Authors:** Elisa Perez-Moreno, Claudia Oyanadel, Adely de la Peña, Ronny Hernández, Francisca Pérez-Molina, Claudia Metz, Alfonso González, Andrea Soza

**Affiliations:** 1 Centro de Biología Celular y Biomedicina (CEBICEM), Facultad de Medicina y Ciencia, Universidad San Sebastián, Santiago, Chile; 2 Centro Científico y Tecnológico de Excelencia (CCTE) Ciencia y Vida, Santiago, Chile; 3 Departamento de Ciencias Biológicas y Químicas, Facultad de Medicina y Ciencia, Universidad San Sebastián, Santiago, Chile

**Keywords:** EMT, Galectin, Cancer, Metastasis, Tissue repair, Fibrosis, Epithelial-mesenchymal plasticity

## Abstract

Galectins are soluble glycan-binding proteins that interact with a wide range of glycoproteins and glycolipids and modulate a broad spectrum of physiological and pathological processes. The expression and subcellular localization of different galectins vary among tissues and cell types and change during processes of tissue repair, fibrosis and cancer where epithelial cells loss differentiation while acquiring migratory mesenchymal phenotypes. The epithelial-mesenchymal transition (EMT) that occurs in the context of these processes can include modifications of glycosylation patterns of glycolipids and glycoproteins affecting their interactions with galectins. Moreover, overexpression of certain galectins has been involved in the development and different outcomes of EMT. This review focuses on the roles and mechanisms of Galectin-1 (Gal-1), Gal-3, Gal-4, Gal-7 and Gal-8, which have been involved in physiologic and pathogenic EMT contexts.

## Background

Galectins are carbohydrate-binding proteins that regulate a variety of cellular processes by interacting with β-galactoside moieties present in glycoproteins and glycolipids [1–6]. Galectins are found in the cytosolic compartment and extracellularly after unconventional secretion [3, 6]. In contrast with the regulation systems mediated by ligand binding to a specific cell surface receptor, galectins globally contribute to modulate these regulation systems through their simultaneous interaction with different signaling receptors [1, 3–6]. As the structures conformed by glycans entail enormous versatility and are considered to constitute a “sugar code” susceptible to changes under physiological or pathological conditions [1, 2], galectin functions also display variations accordingly with the glycan modifications [4, 7–9]. Physiological processes such as cell proliferation, differentiation, phenotypic plasticity, apoptosis, angiogenesis and immune responses, as well as pathogenic conditions such as cancer, tissue fibrosis, chronic inflammation and autoimmune disorders, usually engage distinct members of the galectin family with complementary or redundant roles, acting across various tissues and cell types, such as endothelium, epithelium and immune cells [3–6, 10, 11].

This review is not only complementary but also extends the analysis of previous reviews to cover concepts related to mechanisms, regulation systems and cell biology processes known to be involved in the EMT, which have not usually been addressed at the same time or with this conceptual framework [12]. We also call the attention on works where the effect of galectins can be attributed to intracellular or extracellular actions, which is not explicitly addressed by other reviews. Also, we covered a more widely spectra of galectins compared with other reviews that focused on particular sets of galectins. After a general description of glycosylation, galectin secretion, and EMT processes, we organized the literature in separate sections devoted to tissue repair, fibrosis or cancer, emphasizing the role attributed to EMT and the possible contribution of different galectins. This systematic approach illustrates most of the examples where galectins directly or indirectly regulate EMT, including the phenotypic changes and the signaling pathways involved. We hope this approach will provide a more comprehensive understanding of how galectins impact the cell biology of epithelial cells.

### Galectins: structural features as carbohydrate-binding proteins

**Fig. 1:**
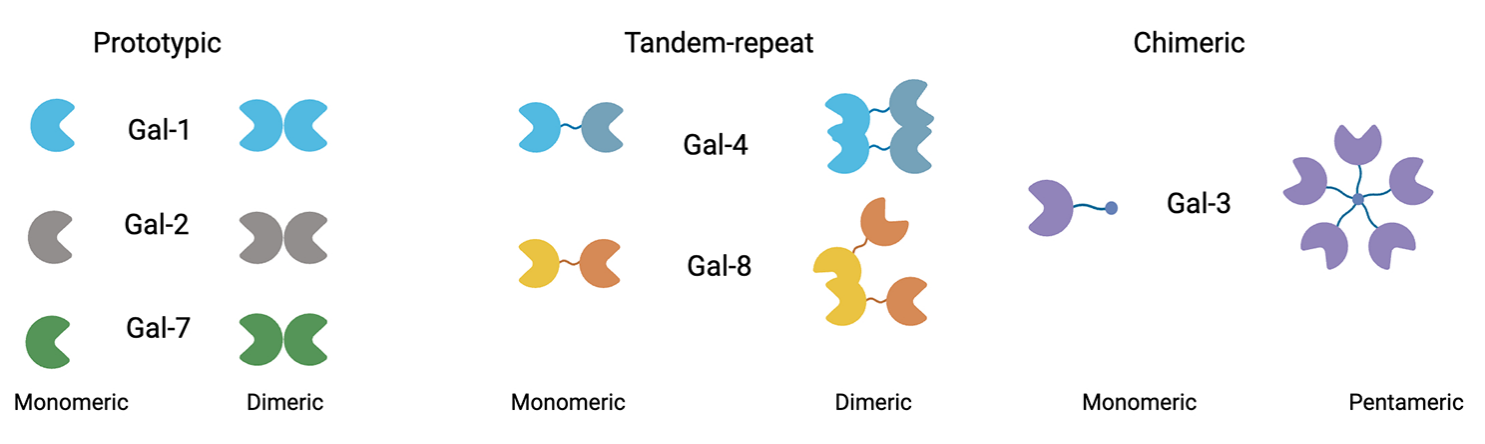
Structure of galectins involved in EMT. Gal-1, Gal-2, and Gal-7 are prototypic galectins that contain a single CRD and a short N-terminal sequence that enables dimerization. Gal-4 and Gal-8 are tandem repeat galectins bearing N- and C-terminal CRDs separated by a linker peptide. These galectins also form dimers. Gal-3 belongs to the chimeric galectin type and contains a single CRD and a large amino-terminal non-lectin domain that contributes to self-aggregation

The members of the galectin family have one or two carbohydrate-binding domains (CRDs) of approximately 130 amino acids conforming a β-sandwich binding groove for β-galactosides [4, 13, 14]. β-galactosides are formed by Galβ (1–4)GlcNAc moieties, also known as N-Acetyllactosamine (LacNac), found with variable configurations in glycoconjugates of N- and O-glycosylated proteins and glycolipids as part of the glycome or glycan code [2, 4, 15]. Sixteen galectins have been described in animals and are classified into three groups based on the number and organization of their CDRs and other structural features [16]. The prototypical galectins (Gal-1, -2, -5, -7, -10, -11, -13, -14, -15, and -16) contain a single CRD and a short N-terminal sequence that allows for dimerization. The tandem-repeat galectins (Gal-4, -6, -8, -9 and -12) contain two different CRDs separated by a linker peptide within a single polypeptide molecule. The chimeric galectin type is only represented by Gal-3 that has a single CRD and a large amino-terminal non-lectin domain that contributes to self-aggregation [3, 16] (Fig. 1).

**Fig. 2:**
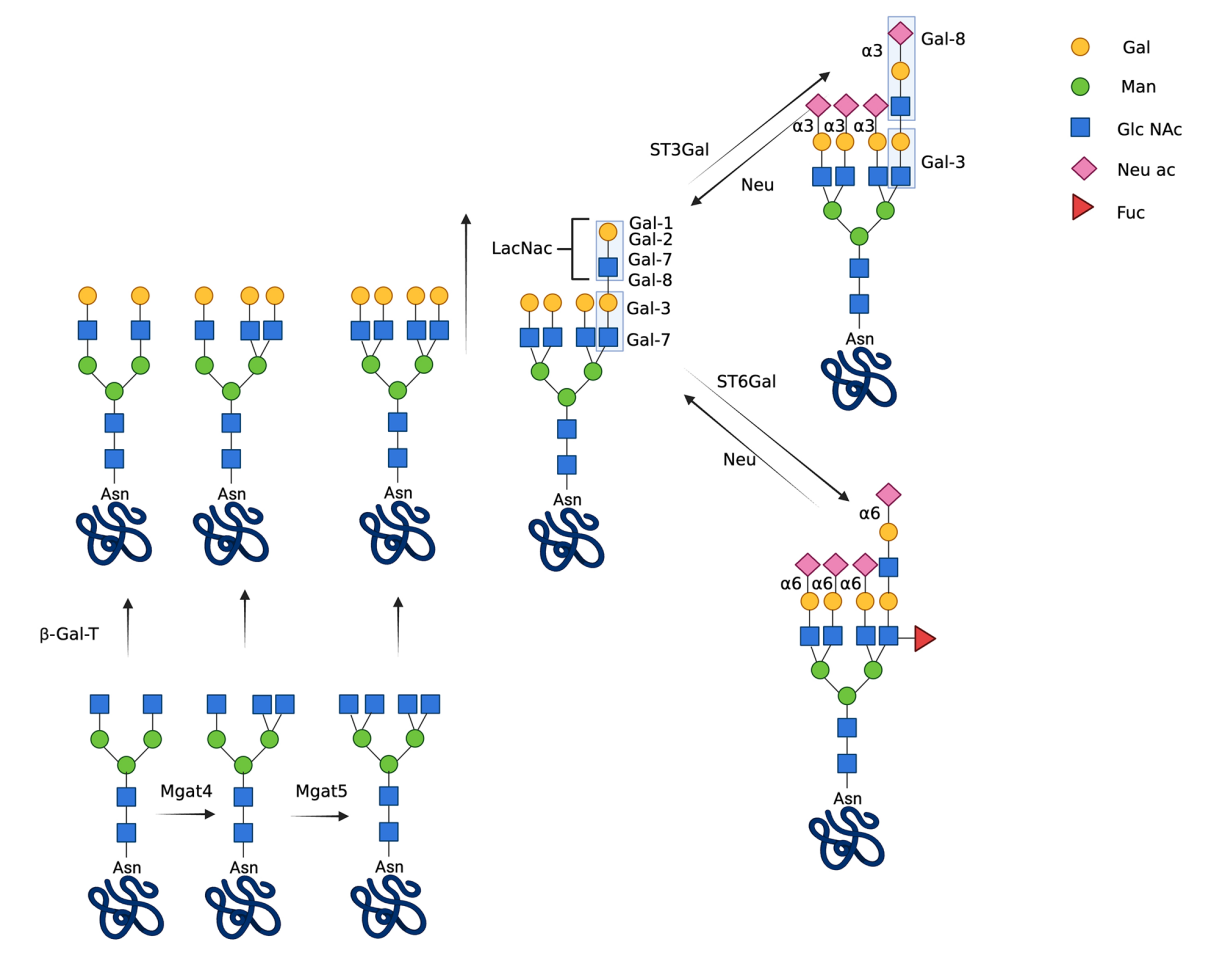
Complex N-linked oligosaccharides with variations in length, branching and sialylation of their LacNAc moieties. The scheme shows some steps of the biosynthesis of complex N-linked oligosaccharides highlighting the role of enzymes that generate variations of N-acetyllactosamine (LacNac) glycoconjugates, the preferred ligand of galectins. N-acetylglucosaminyltransferases (Mgat4 and 5) act sequentially in ramifications; galactosyltransferases (GalTs) catalyze the transfer of galactose to glycoprotein-bound N-acetylglucosamine forming LacNac of variable lengths; α2,6 sialyltransferase 1 (ST6Gal1) adds sialic acid in α2,6 linkages to terminal LacNac, preventing the binding of several galectins, except Gal-3 that also bind to internal LacNac; N-acetylglucosamine α2,3 sialyltransferase 1 (ST3Gal1) adds sialic acid to N-glycans in position α2,3, favoring the binding of Gal-8; fucosyltransferase-3 (Fut3) adds fucose to N-acetylglucosamine; Neuroaminidases (Neus) remove sialic acid affecting the binding of galectins

Although all galectins have affinity for LacNac they show different preferences for LacNac variants displayed by N- and O-glycans or glycolipids [2, 4, 10, 15, 17–20]. Variations of LacNac of N-glycosylated proteins affecting the affinity and function of galectins include differences in N-glycan tree ramifications, LacNac extensions, sialylations or fucosylations, depending on the expression levels and activities of specific Golgi-resident glycosyl-transferases [4, 7, 21, 22]. Another source of LacNac variations that switch the preferential binding of particular galectins is the removal of sialic acid from cell surface glycans mediated by secreted neuraminidases [23–25] (Fig. 2). All these changes have an impact on glycome and are sensitive to a variety of physiological and pathogenic conditions, including inflammation and cancer [4, 7, 21, 22, 25].

Galectins lack a signal peptide and therefore are synthesized in the cytosol and reach the extracellular space after non-conventional secretion [3, 26–31]. In the cytosol, galectins recognize host glycans in damaged endolysosomes and promote their reparation or removal and replacement in response to diverse stimuli [24, 32–36]. Galectins can establish direct protein-protein interactions through their CRD [37], thus also playing carbohydrate-independent functions in diverse cellular processes, including mRNA splicing and stabilization, cell proliferation, apoptosis, and cell cycle progression [38–41]. Once secreted, galectins interact with glycosylated proteins and lipids at the cell surface, thus modulating the activity of signaling elements or modifying membrane properties with consequences over cell proliferation, migration, differentiation, cell-cell or cell-matrix interactions and endocytosis [3, 7, 42].

Numerous proteins, including galectins [43], which lack a signal peptide for the classical exocytic pathway, are nevertheless found in the extracellular media reflecting their sorting towards one or several unconventional secretion pathways [44–47]. In general, unconventional secretion of proteins proceeds through direct translocation across the plasma membrane or through their loading into diverse vesicular vehicles [44]. One of the most studied direct pathways involves a translocation pore conformed by the protein gasdermin after its inflammasome-depending processing [48]. On the other hand, vesicles involved in unconventional secretion either bud from the cell surface or are intracellularly generated at different compartments, including the endoplasmic reticulum-Golgi intermediate compartment (ERGIC), autophagosomes and multivesicular bodies, which then fuse with the plasma membrane and release their contents to the media [44, 45, 47, 49]. The ERGIC pathway includes a heat-shock protein 90-mediated unfolding step occurring at the cytosol followed by a TMED10 translocation system into the ERGIC lumen and the generation of transporting vesicles that then may eventually fuse directly with the plasma membrane or use the autophagosome or the multivesicular body secretion pathways [45, 47]. The evidence suggests that galectins can follow any of these pathways depending on the cell type and particular conditions. For instance, Gal-3 has been found secreted in exosomes corresponding to the intraluminal vesicles of multivesicular bodies from cancer cells, dendritic cells and macrophages [46], as well as from apical-basolateral polarized epithelial cells, being mainly secreted in apical exosomes from MDCK cells [46]. An exosomal independent pathway involving direct gasdermin-mediated plasma membrane translocation has been described in the Gal-3 secretion pathway of primary bone marrow–derived macrophages and dendritic cells [50]. Other galectins found in secreted exosomes include Gal-1 and Gal-9 [31, 46, 51]. Gal-1 can be also secreted through an autophagic secretion pathway [52] and together with Gal-3 have been found secreted through ERGIC-derived vesicles [47]. Furthermore, O-GlcNAcylation (O-linked-betaN-Acetylglucosamine), a post-translational modification, affects the secretion levels of several galectins relating this process to metabolism [53, 54]. All this suggests variations in the mechanisms of galectin unconventional secretion, which may be sensitive to different cellular conditions, with physiologic and pathogenic consequences remaining to be elucidated.

The expression and subcellular localization of galectins depend on individual cell types and are modified in response to physiological conditions, such as tissue repair [55], or pathological conditions such as fibrosis and cancer [1, 4], in which EMT plays an important role [15, 56, 57]. Galectins involved in EMT so far include Gal-1, -2, -3, -4, -7, and Gal-8 (Fig. 1). The roles of these galectins complement or overlap each other depending on the preferences for β-galactoside contexts and their capacity to oligomerize or multimerize [4, 58] (Figs. 1 and 2).

### Epithelial-mesenchymal transition (EMT)

EMT is a dynamic and reversible process whereby epithelial cells acquire variable grades of a mesenchymal phenotype [59]. The diversity of epithelial changes is illustrated by a consensus list of core events as indicators of EMT [59]. This includes cytoskeleton remodeling, loss of apico-basal polarity, acquisition of a front-back polarity, weakening of cell-cell adhesion, remodeling of cell-matrix interactions, cell individualization and acquisition of cell motility [59–61]. EMT involves gene expression changes mainly controlled by Snail, Slug, ZEB and Twist as typical EMT transcription factors [62]. The EMT program most frequently leads to intermediate phenotypes known as “partial EMT” and rarely progresses to a fully mesenchymal state [59, 61, 63]. Partial EMT is characterized by epithelial markers such as E-cadherin, claudin, occludin, and ZO-1, commonly coexisting with mesenchymal markers such as vimentin, α-SMA, N-cadherin and fibronectin [59, 61, 63]. EMT can occur in physiological and pathological processes and has been classified into type 1, type 2 and type 3 EMT.

Type 1 EMT occurs during embryonic development and is important for organ formation, reverting through a mesenchymal-epithelial transition (MET) process to generate secondary epithelia. Type 2 EMT is observed in tissue repair, wound healing, tissue regeneration, fibrosis, and inflammation, where transition back to the original epithelial state normally occurs once the tissue repair is completed or the associated inflammation is reduced [60]. Tissue repair is a multistage dynamic process that includes an inflammatory response, increased cell proliferation, migration, and remodeling of extracellular matrix (ECM) components, with variations in different tissues and pathogenic conditions. For instance, in the skin, epidermal keratinocytes undergo partial EMT, detach, proliferate and move across a provisional matrix in the wound [64–67], which is then replaced by a novel matrix of collagen fibers, proteoglycans, and fibronectin, while keratinocytes re-epithelialize the damaged tissue [68]. EMT-transformed keratinocytes also differentiate to contractile myofibroblasts that physically contract the wound by bringing the injured edges closer [66]. In the kidney, after an acute injury, the remaining tubular epithelial cells participate in the repair process undergoing partial EMT, losing their polarized phenotype and acquiring mesenchymal traits associated with cell proliferation and migration into the damaged zone [69, 70]. A reverse process of MET completes the repair process restoring the original epithelial phenotype [71]. An intriguing aspect is that after damage, renal epithelial cells undergo epithelial dedifferentiation without engaging in the processes of delamination or invasion, but instead remaining attached within the tubules. This distinct behavior of the epithelial cells in the injured kidney highlights the complexity and specificity that repair mechanisms acquire in different organs [69, 70].

Prolonged or uncontrolled type 2 EMT is associated with the activation of fibroblasts towards the production of ECM proteins and the consequential progression to tissue fibrosis [66, 72, 73]. During normal tissue repair, myofibroblasts appear transiently and are then lost by apoptosis, whereas in pathological wound healing myofibroblast activity persists driving tissue alterations and promoting fibrosis [66, 73–75]. The origin of myofibroblasts is still unclear. In different organs, myofibroblasts have been proposed to originate from the activation of resident fibroblasts, differentiation from bone marrow precursors or transformation of epithelial cells through EMT [60]. In the kidney, recent studies in the context of fibrosis reveal that EMT does not directly generate myofibroblasts but rather promotes an arrest of the tubular epithelial cell cycle at the G2/M phase, which triggers a pro-inflammatory secretome, thereby activating neighbor cells and enhancing an immune infiltration that contributes to fibrosis progression [72, 76, 77]. The specific molecular mechanisms and signaling pathways that associate EMT to normal tissue repair or pathogenic fibrosis remain unknown and indeed are important to elucidate to find therapeutic targets for the prevention or treatment of fibrotic diseases [78].

Type 3 EMT occurs in cancer, where transformed epithelial cells lose their epithelial attachments and invade the basement membrane and the surrounding tissue, thus promoting cancer dissemination and metastasis [60, 66]. Metastasis is the leading cause of cancer-associated deaths and includes cell dissemination from the primary tumor and formation of a distal secondary tumor [79]. Loss of E-cadherin and cytokeratin, as well as acquisition of vimentin, N-cadherin and α-SMA, have been found in different types of cancer associated with metastasis [80, 81].

A similar outcome of all three EMT types is that epithelial cells lose differentiation markers, such as the apical-basolateral polarity typical of transporting and secreting epithelia, and in general adopt a mesenchymal phenotype with migratory properties [60]. However, the discovery of new features in a continuous range of hybrid EMT phenotypes, rather than a simple binary epithelial-mesenchymal model, has broadened the definition of EMT by introducing the term *epithelial-to-mesenchymal plasticity* (EMP) [59]. Cells can migrate individually or collectively depending on the degree of cell-cell adhesion loosening. During collective migration, as in partial EMT, cells retain cell-cell interactions and move coordinately in the same direction with higher efficiency compared with individual migration [82, 83]. Collective cell migration occurs in all types of EMT and therefore can be observed during processes of wound healing, fibrosis and cancer metastasis [82, 84, 85]. In cancer-associated collective migration, cells remain attached due to weakened junctions and achieve a coordinated movement that enhances the efficiency of dissemination and metastasis formation [83, 86–88].

Inducers and modulators of the EMT program are intensively studied in non-tumoral and carcinoma cells in different contexts. Secreted factors within the cell microenvironment, such as hepatocyte growth factor (HGF), fibroblast growth factor (FGF), epidermal growth factor (EGF), transforming growth factor β (TGF-β) and Wnt play important roles in EMT acting as paracrine or autocrine stimuli of specific cell surface receptors and signaling pathways [89, 90]. ECM elements can also induce EMT by triggering integrin-mediated signaling [91, 92]. Activation of signaling pathways converges upon the activation of EMT-transcription factors, which downregulate epithelial and upregulate mesenchymal markers [90, 93]. The extent of EMT depends on the cooperation between signaling pathways emerging from different receptors [90, 93].

### EMT and glycosylation changes that affect galectin binding and function

EMT includes changes in the glycosylation patterns characterized by hyper- or hypo-glycosylation of proteins and lipids, or N-glycan remodeling of glycoproteins along the exocytic pathway [12]. As the affinity of different galectins varies depending on glycan modifications, the glycosylation patterns associated with EMT are expected to improve or reduce the binding and function of galectins. Galectins studied in EMT include Gal-1, -2, -3, -4, -7, and  -8 (Fig. 1), playing complementing or overlapping roles based on their preferences for β-galactoside-containing structures [4, 58] (Fig. 2). For instance, cell surface sialylation mediated by ST3Gal α2,3-sialyltransferases, a modification that can improve the binding of certain galectins such as Gal-8 [19], while decreasing the binding of other galectins, such as Gal-2, Gal-3, Gal-4 N, and Gal-7 [94], increases cellular migration and metastasis formation in pancreatic adenocarcinoma cell lines [95]. The enzyme ST6Gal-I mediates LacNac terminal α2,6-sialylation, which decreases the binding of several galectins with less effect on Gal-3, presumably because this galectin also recognizes internal LacNac [96, 97]. Overexpression of ST6Gal has been found promoting tumor cell survival in many human cancers [96, 97], and has been described to promote EMT in pancreatic cancer cells [98]. Human retinal pigment epithelial cells respond to damage undergoing EMT associated with an increase of acetylglucosaminyltransferase V (Mgat5) activity, which promotes N-glycan ramifications and facilitates the attachment of certain galectins, including Gal-3, to the cell surface [99] (Fig. 2).

### Galectins in tissue repair

Several studies indicate that galectins promote tissue repair suggesting their involvement in the EMT process required for effective repair mechanisms. The role of galectins in tissue repair has been reviewed by Čoma et al. (2023) [100]. However, the direct participation of galectins in EMT-induced tissue repair has not been assessed. Therefore, the specific contribution of galectins to EMT during tissue repair needs to be clarified by dedicated studies. In general, evidence suggests their role may be related to cell migration and re-epithelization facilitating the effects of well-known EMT inducers such as TGF-β or EGF (Table 1).

### Galectin-2 and Galectin-4 in tissue repair

**Fig. 3:**
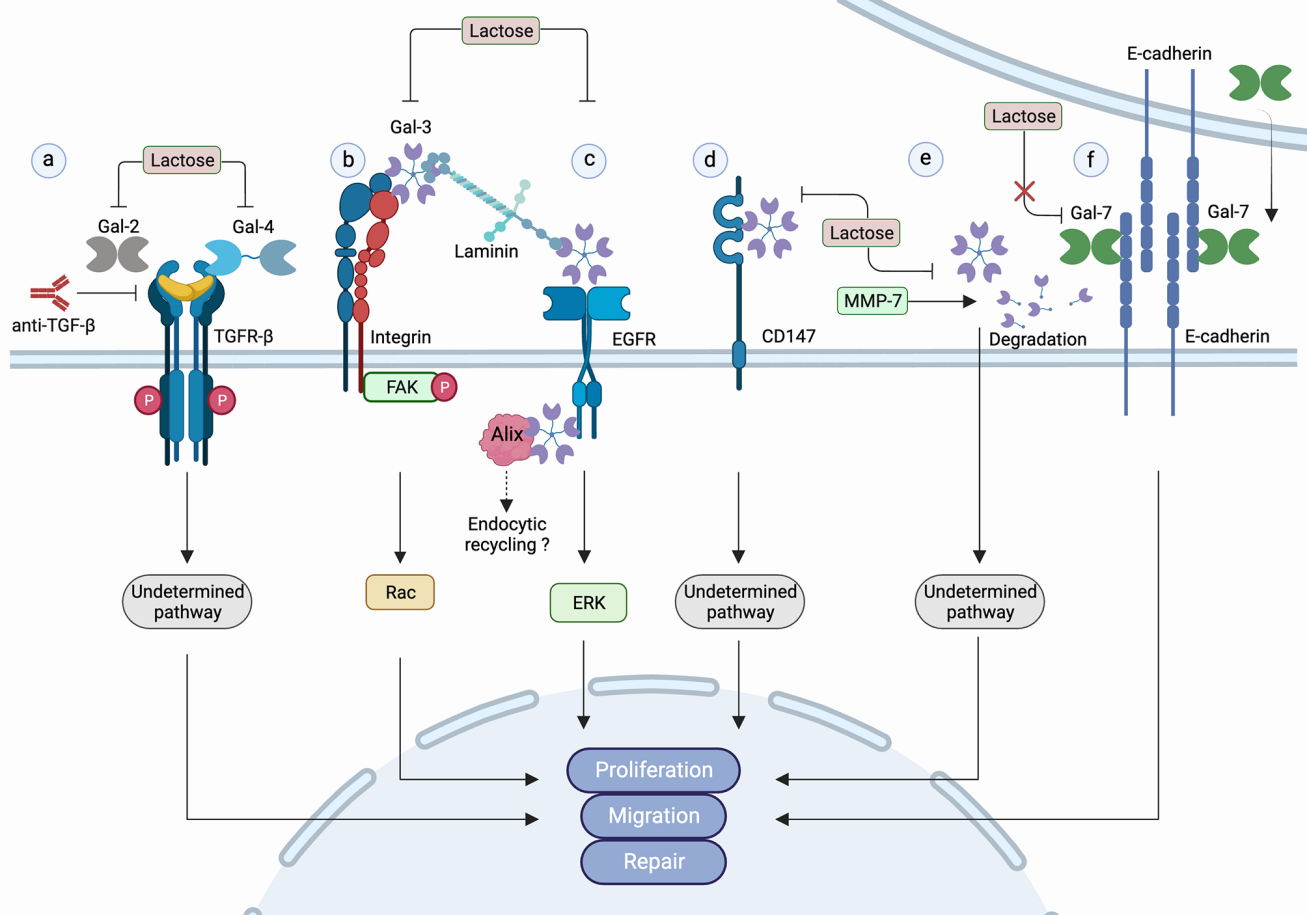
Galectin pathways to tissue repair involving EMT. (**a**) Gal-2 and Gal-4 promote wound healing very likely involving glycan-mediated cell surface interactions and TGF-β-mediated signaling, as their effects are blocked by lactose and antibodies against TGF-β; (**b**) Gal-3 activates the α3β1 integrin/FAK/Rac1 pathway in a carbohydrate-dependent manner to promote re-epithelialization; (**c**) Gal-3 promotes the formation of β4-integrin/laminin332/EGFR clusters leading to ERK signaling activation, enhancing cell migration in a carbohydrate dependent manner. It also links EGFR to ALIX function that may be involved in EGFR pathway to endocytic recycling; (**d**) Extracellular Gal-3 binds to CD147 promoting detachment of retinal pigment epithelial cells from the epithelia in a carbohydrate-dependent manner through an unknown signaling pathway; (**e**) Matrix metalloproteinase MMP-7 can control Gal-3 function through degradation, as shown in wound closure experiments with colon cancer epithelial cells; (**f**) Gal-7 interacts with E-cadherin stabilizing its location on the plasma membrane and favoring collective cell migration in a carbohydrate-independent manner, as shown by the lack of lactose blocking effect

Gal-2 and Gal-4 are specifically expressed in the gastrointestinal tract and both ameliorate experimental colitis when exogenously added [101, 102]. In vitro, Gal-2 and Gal-4 promote wound healing of Caco-2 cell line increasing cell migration. This involves cell surface carbohydrate binding and TGF-β signaling, as their effects are counteracted by lactose-mediated blockage and TGF-β neutralizing antibodies (Fig. 3a) [103]. Even though not directly assessed, these observations suggest that Gal-2 and Gal-4 very likely induce an EMT process in Caco-2 cells. Inflammatory bowel disease and other intestinal disorders that weaken the integrity of the epithelial barrier in conditions such as peptic ulcers, intestinal infections, bowel perforation and many other diseases [104] may involve the function of these galectins.

### Galectin-3 in tissue repair

Gal-3 has been shown to promote re-epithelialization of corneal wounds in mice by interacting with N-glycans present on α3β1 integrin and activating the downstream Focal adhesion Kinase (FAK)/Rac1 pathway (Fig. 3b) [105, 106]. In the absence of Gal-3, corneal re-epithelialization is less efficient due to a reduced secretion of Matrix Metalloproteinase-9 (MMP-9) and impaired cell migration [105, 107]. As re-epithelialization of corneal wounds is not restored by recombinant Gal-3 treatment in Gal-3-KO mice, it is possible that an intracellular function of Gal-3 promotes the healing of corneal wounds [105].

Keratinocytes derived from Gal-3-KO mice (Lgals3^−^/^−^) show reduced cell migration together with lower surface levels of epidermal growth factor receptor (EGFR) most probably due to intracellular trafficking defects, considering that cytosolic Gal-3 mediates the association of EGFR with Alix, a component of the Endosomal Sorting Complex Required for Transport Machinery (ESCRT) [108]. Alix has been involved in a recycling pathway of EGFR [109, 110]. Additionally, extracellular Gal-3 interaction with EGFR contributes to the formation of a cluster with β4 integrin/laminin332 resulting in Extra Cellular Signal Kinase (ERK) activation (Fig. 3c), decreased cell adhesion, enhanced cell migration and wound healing in vitro [111]. These observations suggest that Gal-3 may influence the role of keratinocytes in wound healing modulating the role of EGFR in this process.

In retinal pigment cells, cell migration is promoted by Gal-3 through a glycan-dependent interaction with CD147 (Fig. 3d), a type I transmembrane protein [112]. This interaction with CD147 triggers the secretion of MMP-9, disrupts occludin localization in intercellular junctions and leads to the reorganization of the actin cytoskeleton, resulting in destabilization of cell-cell interactions and detachment of retinal pigment epithelial cells [112]. This effect very likely reflects the role of Gal-3 in the EMT of these cells.

Gal-3 also enhances wound closure in colon cancer epithelial cells involving cell surface carbohydrate interactions (Fig. 3e) [113]. This effect is impaired by recombinant MMP-7 that cleaves endogenously secreted Gal-3 [113], suggesting a relationship with the observation that patients with chronic intestinal diseases have increased levels of MMP-7 [114], and decreased expression of Gal-3 [115]. Cleavage of endogenous Gal-3 by MMP-7 might hinder the process of tissue repair in colon epithelial cells.

### Galectin-7 in tissue repair

Gal-7 overexpression or deficiency has been shown to delay wound healing in mice [116, 117]. Gal-7 overexpression in skin is associated with increased apoptosis and decreased levels of E-cadherin, which also mislocalizes together with β-catenin, resulting in disrupted cohesion of keratinocytes after damage [117]. Gal-7 interacts with the extracellular domain of E-cadherin in keratinocyte cell lines, thus stabilizing its location on the plasma membrane in a carbohydrate-independent manner (Fig. 3f) [117, 118]. Mice lacking Gal-7 expression have an impaired regenerative response due to enhanced apoptosis and decreased migration of keratinocytes [116]. Gal-7-depleted cells show defective collective cell migration [118]. Therefore, Gal-7 role in tissue repair may be exerted through a mechanism involving E-cadherin retention at the plasma membrane and collective cell migration in keratinocytes.

**Table 1:** Summary of the promotion of EMT by Gal-2, Gal-3, Gal-4, and Gal-7 in tissue repair

Galectin	Model	Strategy	EMT phenotype	Signaling pathway	Ref
**Gal-2 and Gal- 4**	Intestinal epithelium cells	Exogenously added, blockade with lactose	Increased wound closure and proliferation	TGF-β	[103]
**Gal-3**	Cornea cells from mice	Deletion in mice, exogenously added, blockade with lactose	Increased wound closure	N.D	[105]
	Colon epithelium cells	Exogenously added, blockade with lactose	Increased wound closure	N.D	[113]
	Mice epidermis and cells	Deletion in mice	Increased cell migration	EGFR/ERK	[108]
	Immortalized keratinocytes and keratinocytes isolated from patients	Exogenously added, blockade with lactose	Weakening of cell-cell adhesion and increased cell migration	β4 integrin/laminin332/EGFR	[111]
	Human corneal keratinocytes and mice	Deletion in mice, exogenously added, blockade with lactose	Occludin mislocalization, weakening of cell-cell adhesion, reorganization of the actin cytoskeleton, MMP-9 secretion, and cell detachment	CD147	[112]
**Gal-7**	Mice	Deletion in mice	E-cadherin/ β -catenin mislocalization, increased proliferation, decreased cell migration	N.D	[116]
	Mice and keratinocyte cell lines	Overexpression in mice epidermis	Decreased E-cadherin, weakening of cell-cell adhesion	N.D	[117]
	Epidermal cells	Knockdown, exogenously added, blockade with lactose	Cell polarity, wound closure, E-cadherin on cell surface	N.D	[118]

### Galectins and EMT in fibrosis

Fibrosis is characterized by an excess of proteins of the ECM leading to scar tissue development, organ dysfunction and failure [15]. Studies regarding the role of galectins in fibrosis by promoting EMT have mainly been focused on Gal-1 [119–121] and Gal-3 [122–125] (Table 2).

### Galectin-1 in EMT of fibrosis

**Fig. 4:**
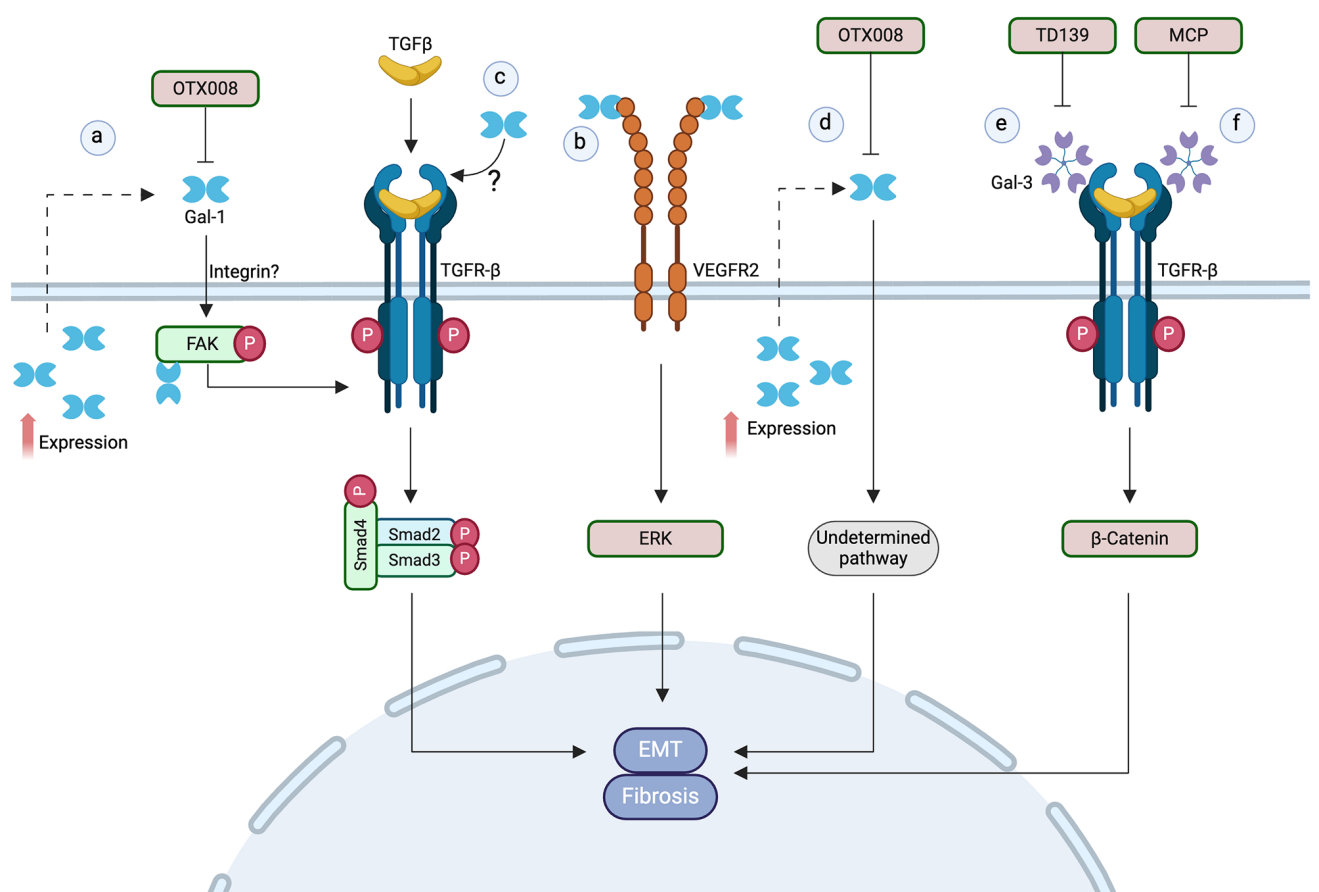
Gal-1- and Gal-3 pathways promoting EMT in fibrosis. (**a**) Gal-1 promotes lung and sub-retinal fibrosis involving activation of a FAK/TGFR-β/Smad pathway, which in turn induces Gal-1 expression as positive feedback and is counteracted by Gal-1 inhibitor OTX008. This pathway most likely includes activation of β1-integrins upstream FAK. Intracellular Gal-1 interacts with FAK1 suggesting an additional intracellular unknown role. Indeed, TGFR-β/Smad can be directly stimulated by TGF-β with similar effects. In retinal epithelial cells Gal-1 activates VEGFR2-ERK to promote subretinal fibrosis (**b**) or can enhance the EMT phenotype induced by TGF-β/Smad signaling (**c**). (**d**) Gal-1 expression is enhanced in a cellular model of diabetic retinopathy promoting EMT-associated fibrosis through an unknown mechanism, which can be blocked by OTX008. Gal-3 interacts with TGF-βR contributing to the TGF-β1-induced EMT involved in lung and renal fibrosis. The pathway includes β-catenin and can be blocked by Gal-3 inhibitors TD139 (**e**) and MCP (**f**)

In idiopathic pulmonary fibrosis, a progressive and chronic disease, lung tissues overexpress Gal-1 [126]. Mouse models and cell line experiments also support a pro-fibrotic role of Gal-1 in lung fibrosis [119]. When mice are exposed to hypoxia, Gal-1 and α-SMA levels increase associated with lung damage and collagen deposition, effects that are reduced by the Gal-1 inhibitor OTX008 [119]. Hypoxic lung epithelial cell lines show increased cell proliferation and migration, together with expression of pro-fibrotic genes and extracellular matrix proteins. In addition, Gal-1 expression increases under the influence of a FAK/TGF-β/Smad signaling pathway and recombinant Gal-1 treatment promotes FAK activation, which can be blocked with OTX008 to attenuate fibrosis progression (Fig. 4a) [119]. Intracellular Gal-1 binds to and activates FAK1 in these cells, indicating that Gal-1 can activate FAK1 both intracellularly and extracellularly [127].

In subretinal fibrosis induced by choroidal neovascularization through EMT, studies using Gal-1 knockout mice (Lgals1^−/−^) demonstrate that Gal-1 participates in this pathogenic condition by reducing the activation of ERK1/2 pathway, possibly decreasing vascular endothelial growth factor receptor 2 (VEGFR2) signaling (Fig. 4b). In human retinal pigment epithelial (RPE) cells, Gal-1 does not induce EMT but enhances the upregulation of EMT markers induced by TGF-β1/Smad/Snail signaling [120] (Fig. 4c). Although in this model Gal-1 alone may not be sufficient to induce EMT, its silencing prevents the EMT induced by TGF-β1 [120].

Gal-1 has also been implicated in the pathogenesis of diabetic retinopathy, a common complication of diabetes that affects blood vessels in the retina [128]. Exposure of RPE cells to high glucose, emulating the intraocular conditions of patients with diabetic retinopathy, increases the expression of Gal-1 together with mesenchymal markers [121]. In these cells, Gal-1 inhibitor OTX008 not only enhances cell viability but also reduces Gal-1, α-SMA and fibronectin protein levels, ROS production, TGF-β1 mRNA, and the expression of its receptors, TGF-βR1 and TGF-βR2 (Fig. 4d). The evidence suggests that Gal-1 inhibition impairs EMT, preventing its pro-fibrotic effects [121]. Given that Gal-1 is highly expressed in the microenvironment within the eyes of patients suffering from retinopathies [129, 130], its inhibition may offer a therapeutic approach for preventing the visual impairment caused by retinopathies [129–132].

### Galectin-3 in EMT of fibrosis

Gal-3 has emerged as an important player in the pathogenesis of lung fibrosis [122, 133]. Human biopsies and serum from patients with idiopathic pulmonary fibrosis have elevated Gal-3 levels [122, 133]. Mackinnon et al. studies [122] show that inhibition of Gal-3 with TD139 or its deletion in mice reduces lung fibrosis induced by TGF-β1 (Fig. 4e). Primary cultures of WT epithelial alveolar cells (AECs) incubated with TGF-β1 acquire classical mesenchymal traits concomitant with an increased Gal-3 secretion. In contrast, AECs derived from Gal-3^−/−^ mice do not respond to TGF-β1 and maintain their epithelial phenotype. In the same study, Gal-3 silencing in A549 lung cancer epithelial cells reduced the TGFR-β levels at the cell surface and the TGF-β1-mediated activation of β-catenin is restored by Gal-3 treatment (Fig. 4e) [122]. Therefore, Gal-3 is crucial for TGF-β1-triggered EMT involving β-catenin activation [122]. In addition, recent clinical trials show that suppressing Gal-3 expression with TD139 decreases the plasma levels of biomarkers associated with idiopathic pulmonary fibrosis progression [134, 135], suggesting Gal-3 as a promising therapeutic target.

Silica fibrosis, also known as silicosis, is a lung disease caused by inhaling crystalline silica particles, a common mineral found in rocks, sand, and soil [136]. Mice exposed to silica show increased Gal-3 expression, EMT, and the development of lung fibrosis through the activation of the GSK-3β/β-catenin signaling pathway. Knocking down Gal-3 in lung epithelial cell lines or blocking Gal-3 with the inhibitor TD139 (Fig. 4e) in mice prevents EMT and reduces GSK-3β activation and β-catenin translocation to the nucleus, indicating that Gal-3 plays a crucial role in silica-induced EMT [123].

Gal-3 expression is also increased in a rat model of renal damage by spontaneous hypertension [124]. Treatment with modified citrus pectin (MCP) (Fig. 4f), a Gal-3 CRD selective inhibitor that also hinders the expression of Gal-3, reduces renal injury markers such as NGAL and Kim-1, as well as fibrotic markers [124]. Gal-3 treatment reduces E-cadherin and β-catenin levels in renal proximal tubular epithelial cells associated with increased mesenchymal markers [124]. Moreover, Gal-3 overexpression enhances the induction of EMT by TGF-β1, suggesting that extracellular Gal-3 contributes to TGF-β1-induced renal fibrosis through EMT [125].

**Table 2:** Summary of Gal-1- and Gal-3-promoted EMT in fibrosis

Galectin	Model	Strategy	EMT Marker	Signaling Pathway	Ref
**Gal-1**	Pulmonary fibrosis induced by hypoxia in mice and cells	Inhibition with OTX008	Collagen, Fibronectin, Metalloproteinases, TGF-β	FAK1/TGF-β/Smad	[119]
	Subretinal fibrosis in cells and mice	Deletion in mice and cells, overexpression in mice and cells	TGF-β1, α-SMA, Type I collagen, fibronectin, Snail	VEGFR2/ERK; TGF-β1/Smad2/Snail	[120]
	Diabetic retinopathy in cells	Inhibition with OTX008	TGF-β, α-SMA, fibronectin	TGF-β	[121]
**Gal-3**	Pulmonary fibrosis in mice and cells	Knockout mice, knockdown and exogenously added in cells, blockade with TD139	E-cadherin, α-SMA, morphologic shape	TGF-β-mediated β-catenin activation	[122]
	Silica-induced pulmonary fibrosis in mice and lung carcinoma epithelial cells	Blockade in mice and cells with TD139, knockdown in cells	E-cadherin, vimentin, α-SMA	β-Catenin	[123]
	Renal fibrosis in cells and spontaneously hypertensive rats	Blockade in mice, exogenously added in cells, blockade with MCP	E-cadherin, Type I collagen, Type IV collagen, fibronectin, vimentin, α-SMA, TGF-β, CTGF	β-Catenin	[124]
	Renal fibrosis in cells	Knockdown, overexpression	E-cadherin, α-SMA, vimentin, fibronectin, MMP-9	AKT/GSK3β/Snail	[125]

### Galectins in cancer and EMT

EMT has been associated with cancer progression promoting malignant traits such as stemness [137], chemo-resistance [138, 139], mortality [140] and metastasis [60]. Metastasis is the leading cause of cancer-associated deaths and includes cell dissemination from the primary tumor and formation of a distal secondary tumor [79]. Numerous studies describe the function of certain galectins in cancer [15, 141–143], acting upon cell proliferation, attachment to extracellular-matrix and angiogenesis [11, 15, 42]. Beyond their role in angiogenesis, which has been recently reviewed [11, 15, 42], it is possible that galectins also enhances the permeability of the endothelium, a crucial phenomenon in tumor microenvironment, as suggested by studies on Gal-8 [144]. However, the role of galectins in EMT promotion is less understood and has largely been restricted to Gal-1 [145–148], Gal-3 [149–151], Gal-4 [152], and Gal-8 [153] (Tables 3 and 4).

### Galectin-1 in EMT associated with cancer

**Fig. 5:**
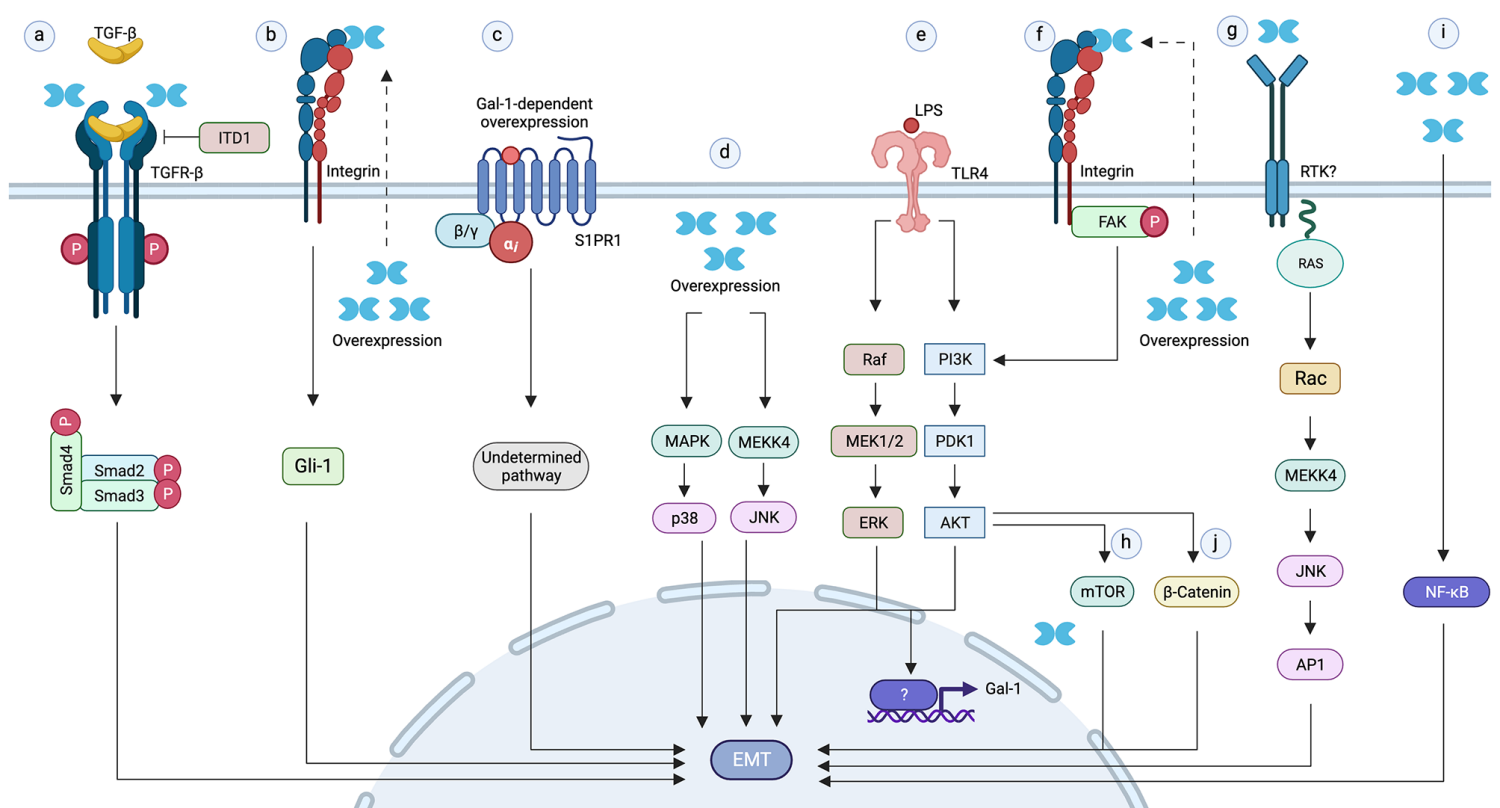
Gal-1 pathways promoting EMT in cancer. (**a**) Gal-1 promotes EMT in gastric cancer cells through a TGFR-β/Smad pathway and can be blocked with the Gal-1 inhibitor ITD1. (**b**) Gal-1 secreted from gastric cancer cells or CAFs induces EMT through a mechanism involving β1-integrin and Gli-1. (**c**) Overexpression of Gal-1 in gastric cancer cells increases the levels of S1PR1 at the cell surface, which is required for Gal-1-induced EMT through an unknown pathway. (**d**) Overexpression of Gal-1 in ovarian cancer cells activates MAPK JNK/p38 signaling through an unknown mechanism promoting EMT. (**e**) Activation of TLR-4 induces Gal-1 via PI3K/AKT or ERK-AKT pathways leading to EMT. (**f**) Gal-1 overexpression in HCC cells induces EMT through a Integrin/FAK/PI3K/AKT pathway. (g-j) Examples of extracellular Gal-1 contribution to EMT, involving Ras/Rac1/MEKK4/JNK/AP (**g**) and FAK/PI3K/AKT/mTOR (**h**) in urothelial cancer cells, activation of NFkB signaling in pancreatic cancer cells (**i**) and activation of β -catenin pathway in colorectal cancer (**j**)

In gastric cancer, increased levels of Gal-1 have been associated with lower overall and disease-free survival, as well as with an increased incidence of lymph node metastasis in patients [154–156]. Gastric cancer tumors have been described to express high levels of Gal-1 associated with low E-cadherin/high vimentin expression [155], indicators of EMT. Gastric cancer cell lines produce Gal-1, which promotes EMT and increases proliferation, invasion and metastatic potential of these cells [155, 157]. Gal-1 overexpression in gastric cancer cells activates the EMT-related TGF-β/Smad signaling pathway (Fig. 5a). This effect is counteracted with the TGF-β inhibitor ITD1, while in turn Gal-1 suppression inhibits TGF-β1-stimulated EMT. These results involve the TGF-β pathway as mediator of Gal-1-induced EMT and indicate that Gal-1 is required for TGF-β1-mediated induction of EMT [155]. Additionally, treatment with recombinant Gal-1 induces Gli-1 expression through a non-canonical hedgehog pathway, which compromises β1-integrin and contributes to EMT independently of the Smoothened receptor (Fig. 5b) [157, 158]. A similar Gli-1-dependent pathway towards EMT is induced by overexpression of Gal-1, which is partially inhibited by lactose treatment indicating dependency of Gal-1 interactions with glycans at the cell surface [157]. Other studies involve the sphingosine-1 phosphate receptor-1 (S1PR1) in EMT associated with cell invasion and metastasis induced by Gal-1 in gastric cancer cells [159]. Overexpression of Gal-1 increases the expression of S1PR1 and enhances cell invasion and metastasis through EMT, while in contrast Gal-1 silencing leads to decreased invasion [159]. The simultaneous knockdown of Gal-1 and overexpression of S1PR1 rescues the invasive ability of cells, demonstrating that Gal-1 promotes EMT via an S1PR1-dependent mechanism (Fig. 5c). Furthermore, overexpression of Gal-1 and S1PR1 is correlated in gastric cancer tumors [159]. All this suggests that both Gal-1 and S1PR1 may contribute to the invasive properties of gastric cancer cells.

In ovarian cancer, serum samples show that Gal-1 levels are increased and correlate with a higher histological grade and lymph node metastasis [146, 160]. The expression of Gal-1 is inversely correlated with E-cadherin levels in ovarian cancer tissues, suggesting an association between Gal-1 and EMT in this cancer. In ovarian cancer cell lines, Gal-1 overexpression promotes EMT and increases cell migration and invasion through the activation of the MAPK-JNK/p38 signaling pathway, while silencing of Gal-1 has opposite effects (Fig. 5d). Gal-1 overexpression also promotes tumor growth and liver metastasis in mice, favoring the development of tumors with a mesenchymal phenotype [146].

Another receptor that plays a role in EMT is the Toll-like Receptor-4 (TLR-4), which has been associated with metastasis [161–165]. In ovarian and colorectal cancer cell lines, the activation of TLR-4 by lipopolysaccharides (LPS) upregulates Gal-1 expression through the PI3K/AKT pathway, consequently promoting EMT (Fig. 5e) [166, 167]. On the other hand, Gal-1 treatment induces EMT and increases cell invasion involving PI3K and TLR-4 [166], while Gal-1 silencing decreases EMT-related cytokines and suppresses mesenchymal characteristics of LPS-activated colorectal cancer cell lines [167]. Inhibition of ERK phosphorylation downstream TLR4 also reduces Gal-1 expression and prevents EMT [167] (Fig. 5e). All this highlights a critical role of Gal-1 in TLR4-mediated EMT [167].

Gal-1 also promotes the progression of hepatocellular carcinoma (HCC) and upper urinary urothelial carcinoma [127, 168]. Poor recurrence-free and overall survival correlates with Gal-1 expression in HCC patients [127, 168]. Gal-1 overexpression induces EMT in HCC cell lines through the integrin/FAK/PI3K/AKT pathway (Fig. 5f) [127, 148], whereas Gal-1 inhibition suppresses EMT [127]. In urothelial cancer cell lines, Gal-1 enhances invasion by increasing the expression of MMP-9 via the Ras/Rac1/MEKK4/JNK/AP1 pathway (Fig. 5g) [169]. Gal-1 also stimulates the FAK/PI3K/AKT/mTOR pathway (Fig. 5h), which additionally enhances the metastatic behavior of these carcinoma cells [168].

In squamous cell carcinoma (SCC) cells, where EMT has been induced by Snail overexpression, the levels of Gal-1 have been found increased by transcriptional mechanisms involving NF-κB [145]. Gal-1 activates c-Jun and increases the expression of α2 and β5 integrin, enhancing collective cell migration in an autocrine manner [145]. Exogenously added Gal-1 enhances cell invasion together with higher expression of EMT markers only in SNAIL-expressing SCC cells. SNAIL seems to be required for Gal-1 overexpression to potentiate the increment of EMT markers.

Studies conducted with stromal cells, regarding the relevance of the tumor microenvironment in cancer progression and metastasis, reveal an additional Gal-1-mediated EMT [155, 158, 170, 171]. High levels of Gal-1 are detected in stromal cells from gastric cancer and pancreatic ductal adenocarcinoma tumors in correlation with an EMT phenotype of carcinoma cells [155, 158, 170]. Conditioned media from cancer-associated fibroblasts (CAFs) promotes EMT via Gli-1 in gastric cancer cell lines, involving a Gal-1-mediated β1-integrin activation (Fig. 5b) [157, 158]. Gal-1-overexpression in pancreatic stellate cells (PSC) induces EMT in co-cultured pancreatic carcinoma cells, enhancing their proliferation and invasion through the NF-κB pathway (Fig. 5i) [170]. Orthotopic implantation of PANC-1 cells mixed with Gal-1-expressing PSCs promotes tumor growth and liver metastasis in mice [170]. In mouse models of colorectal cancer, both CAF-secreted Gal-1 and recombinant Gal-1 have been shown to activate SOX9 and β-catenin leading to the expression of the EMT inducers Twist and Slug, promoting metastasis (Fig. 5j) [171]. All these studies reveal a crucial role of Gal-1 produced by tumor stromal cells in cancer progression. Indeed, Gal-1 has been considered an interesting therapeutic target in cancer [172].

**Table 3:** Summary of the role of Gal-1 in the promotion of EMT in cancer

Galectin	Model	Strategy	EMT Marker	Signaling Pathway	Ref
**Gal-1**	CAFs derived from gastric cancer patients, gastric cancer cell lines (MGC-803)	CAF secreted, overexpression and knockdown in CAFs	E-cadherin Vimentin	β1-integrin activation	[158]
	Gastric cancer cell line (MGC-803)	Overexpression, knockdown		N. D	[147]
	Gastric cancer cell lines (MGC-803 and SGC-7901)	Overexpression, knockdown, CAF secreted		TGF-β/ Smad	[155]
		Overexpression		S1PR1	[159]
	Gastric cancer cell lines (MGC-803 and MKN-74)	Overexpression, knockdown, exogenously added, blockade with lactose		Gli-1	[157]
	Ovarian cancer cell lines (SK-OV-3 and SK-OV-3-ip)	Overexpression, knockdown	E-cadherin N-cadherin, vimentin, MMP-7, fibronectin, Snail, Slug	MAPK JNK/p38	[146]
	Ovarian cancer cell line (SK-OV-3)	Secretion stimulation with LPS, knockdown	E-cadherin MMP-2, MMP-9, N-cadherin, vimentin, α-SMA, Snail, Slug	TLR4 activation/ PI3K/AKT	[166]
	Colorectal cancer cell lines (HCT116, HCT8 and HT29)	Secretion stimulation with LPS, exogenously added, knockdown	E-cadherin Snail, vimentin, α-SMA, ZEB1, N-cadherin, MMP-2, MMP-9	TLR4/ERK	[167]
	Colorectal cancer cell line (KM12C), fibroblasts cell lines (MRC-5 and WS1)	Knockdown, CAFs secreted, exogenously added	E-cadherin, Twist1, Slug	𝛽-catenin	[171]
	HCC cell lines (Huh7, Hep3B, HCCLM3 and MHCC97H)	Overexpression, knockdown	E-cadherin N-cadherin, vimentin	α5β3-integrin FAK/ PI3K/AKT	[127]
	HCC cell line (HepG2)	Overexpression, knockdown	E-cadherin, ZO-1 Vimentin, Snail	PI3K/AKT/ 𝛽-catenin	[148]
	Upper urothelial carcinoma cell lines (BFTC-909, T24 and J 82)	Exogenously added, knockdown	E-cadherin, ZO-1 Snail, vimentin, N-cadherin, β-catenin, MMP-2, MMP-9	FAK/PI3K/AKT	[168]
	Pancreatic ductal adenocarcinoma cell lines (PanC-1)	Overexpression, knockdown, secreted from stromal pancreatic stellate cells	E-cadherin Vimentin, Twist, MMP-9	NF-κB	[170]

### Galectin-3 in EMT associated with cancer

Gal-3 overexpression has been associated with poor prognosis and lower patient survival rates in different types of cancer [150, 173–178].

**Fig. 6:**
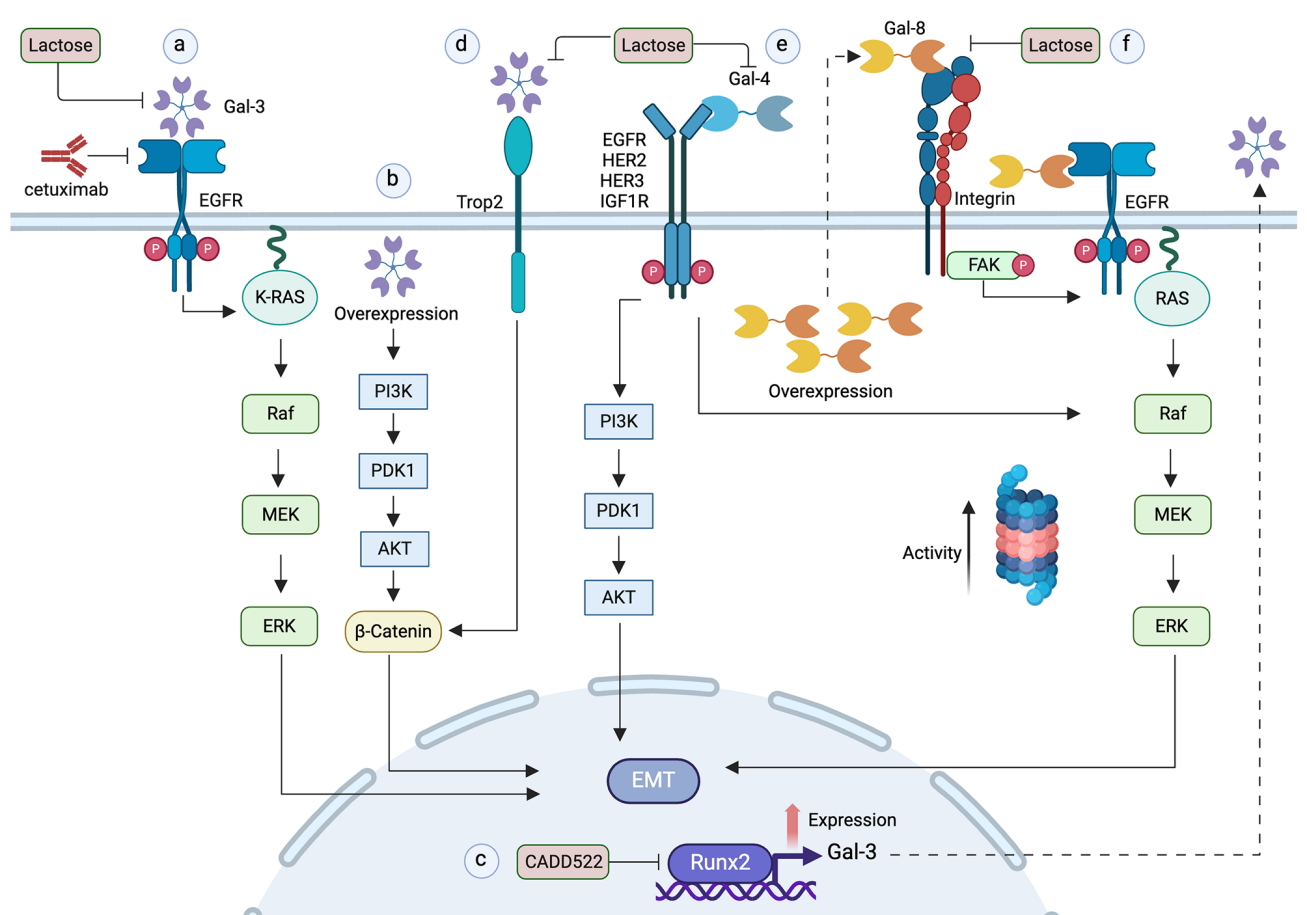
Gal-3, Gal-4 and Gal-8 pathways to EMT in cancer. (**a**) Extracellular Gal-3 promotes cell migration of colon cancer cells in a carbohydrate-dependent manner through a EGFR/K-Ras-Raf-ERK pathway. (**b**) Gal-3 overexpression activates a PI3K/AKT/GSK-3β/β-catenin signaling pathway that induces EMT and promotes metastasis in mice, and is uncertain whether this effect includes secreted Gal-3. (**c**) Runx2 overexpression enhances Gal-3 expression and promotes EMT in hepatocellular carcinoma and lung cancer cells. (**d**) In different cancer cell lines, extracellular Gal-3 interacts with Trop2 in a carbohydrate-dependent manner to promote the nuclear translocation of β-catenin, leading to EMT. (**e**) In prostate cancer, extracellular Gal-4 binds to and activates different tyrosine kinase receptors that trigger ERK and AKT pathways leading to EMT. (**f**) Gal-8 overexpression promotes partial EMT that stimulates cell proliferation, migration, invasion and tumor formation of MDCK cells involving an Integrin/FAK/EGFR pathway and proteasome activation through un unknown mechanism

In colon cancer, increased levels of Gal-3 coincide with decreased E-cadherin expression [150] and treatment of colon cancer cell lines with recombinant Gal-3 promotes EMT characterized by increased cell migration and invasion [150, 179]. The metastatic behavior of colon cancer cell lines is enhanced by Gal-3 in a carbohydrate-dependent manner through the EGFR/K-Ras–Raf–ERK pathway and is blocked by the EGFR inhibitor cetuximab (Fig. 6a) [179, 180]. Downregulation of Gal-3 expression reduces tumor growth in xenograft colon cancer models whereas its overexpression enhances the metastatic potential of cancer cells [180].

In gastrointestinal cancer, inhibition of Gal-3 with low-molecular-weight citrus pectin (LCP), a complex polysaccharide with abundant galactosyl residues, suppresses in vitro cell growth and EMT and inhibits tumor growth through apoptosis and EMT reversion in nude mice xenografts [181].

HCC cell lines, Gal-3 overexpression induces EMT through the PI3K/AKT/GSK-3β/β-catenin signaling pathway and promotes metastatic potential in (Fig. 6b), whereas Gal-3 knockdown suppresses metastasis in mice models [175]. Gal-3 silencing also increases the sensitivity of HCC cells to sorafenib, suggesting a therapeutic potential in patients who develop resistance to this drug [175]. Other studies suggest that Gal-3 mediates the effects of the transcription factor Runx2 [182], which enhances migration and invasion inducing EMT in HCC cell lines [183, 184]. Upregulation of Runx2 increases whereas its knock-down decreases Gal-3 expression [182]. Silencing of Runx2 or inhibition of its DNA binding domain with CADD522 prevents the expression of Gal-3 and EMT, suggesting that Runx2 activation regulates EMT through Gal-3 (Fig. 6c) [185]. Human lung epithelial cancer cells exposed to cigarette smoke extracts, a condition that promotes EMT leading to an enhanced cell invasive migration, also have increased levels of Runx2 and Gal-3 [185]. Epithelial lung cancer cells treated with recombinant Gal-3 shows increased cell invasion, migration, and colony formation reflecting EMT [185].

In oral tongue squamous cell carcinoma cell lines, Gal-3 overexpression promotes EMT whereas its silencing has the opposite effect [149]. Increased Gal-3 levels enhance cell proliferation, migration and invasion through the activation of cannonical Wnt/β-catenin signaling pathway, whereas the Wnt signaling antagonist DKK1 counteracts these Gal-3 effects, thus involving a Gal-3-Wnt-signaling pathway in the promotion of EMT [149].

The role of Gal-3 in breast cancer is controversial. While some publications demonstrate that Gal-3 participates in the promotion of EMT [186, 187], others suggest a protective role [151]. In triple negative breast cancer (TNBC) cells, elevated levels of endogenous Gal-3 correlate with a mesenchymal phenotype, while Gal-3 silencing promotes the reversal process MET together with decreased cell migration [187]. In breast, colon, and prostate cancer cell lines exogenously added Gal-3 promotes EMT by its interaction with Trop-2, a highly-glycosylated membrane protein involved in cancer progression (Fig. 6d) [186]. The mechanism involves phosphorylation and cleavage of Trop-2, nuclear translocation of β-catenin and activation of Zeb1 expression, in a carbohydrate-dependent manner (Fig. 6d) [186].

In contrast with publications from Iwamoto (2023) [186]  and Jeethy Ram (2023) [187], Gal-3 seems to play a protective role against breast cancer malignancy [151]. Advanced locoregional invasion and decreased overall survival of patients with breast cancer correlates with lower expression levels of Gal-3, and Gal-3 silencing in breast cancer cell lines reduces Wnt and Akt signaling, decreases anoikis, promotes EMT, enhances drug resistance and stimulates their tumor growth in mice [151]. The mechanisms of contrasting roles of Gal-3 in different breast cancer cell lines remain unknown.

### Galectin-4 in EMT associated with cancer

Gal-4 has been reported in human prostate cancer tissues with expression levels correlating with metastasis and poor patient survival [152]. In metastatic prostate cancer cell lines, Gal-4 has been shown to bind different receptor tyrosine kinases, such as EGFR, HER2, HER3 and IGF1R, promoting their activation and downstream ERK and AKT signaling promoting EMT in a carbohydrate-dependent manner (Fig. 6e) [152]. Gal-4 seems to be required for cancer progression, as its downregulation decreases tumor growth and lung metastasis in mice [152].

### Galectin-8 in EMT associated with cancer

Gal-8 is a widely expressed galectin in human tissues and carcinomas [28] and has been associated with an unfavorable prognosis in various types of cancer [188, 189]. Gal-8 contributes to cancer progression and metastasis by regulating the production of immunoregulatory cytokines, thereby facilitating the recruitment of cancer cells to metastatic sites [190]. A role of Gal-8 in metastasis has been described in prostate cancer cells, in which Gal-8 silencing decreases E-cadherin levels, migration capabilities and lymph node invasion [189].

In the non-tumoral MDCK cell line, Gal-8 overexpression triggers EMT associated with enhanced cell migration and invasion (Fig. 6f) [153]. The mechanism involves extracellular glycan-dependent interactions with α5β1 integrin followed by FAK activation and EGFR transactivation, as well as proteasomal overactivity characteristic of cancer cells [153]. Gal-8-overexpressing transfected MDCK cells show increased levels of α5β1 integrin, extracellular matrix-degrading MMP13 and urokinase plasminogen activator/urokinase plasminogen activator receptor (uPA/uPAR) protease systems, which very likely contribute to their tumorigenic properties in xenografts experiments in immunodeficient mice [153]. Therefore, Gal-8 has the potential to transform normal epithelial cells into tumoral cells involving EMT [153].

These observations suggest that Gal-8 expression may contribute to carcinoma malignancy [28, 188, 189] by promoting a pro-invasive and metastatic EMT phenotype.

**Table 4:** Summary of Gal-3 and Gal-8 in EMT associated with cancer

Galectin	Model	Strategy	EMT Marker		Signaling Pathway	Ref
			Loss	Increase		
**Gal-3**	Colon cancer cell line (SW480)	Exogenously added	E-cadherin	N-cadherin, vimentin, Twist	N. D	[150]
	HCC cell lines (SMMC 7721, HepG2)	Overexpression, knockdown	E-cadherin	Vimentin	N. D	[182]
	HCC cell lines (Huh7, Hep3Bm, HepG2, SK-Hep1)	Overexpression, knockdown		N-cadherin, vimentin, MMP-1	PI3K/AKT/GSK-3β/β-catenin	[175]
	Gastrointestinal cancer (AGS and SW-480)	Blockade with LCP	E-cadherin	Vimentin, Snail, Twist, ZEB-1	N. D	[181]
	Lung adenocarcinoma cell line (A549)	Exogenous added, blockade with GB117	E-cadherin	N-cadherin, vimentin	N. D	[185]
	Oran tongue squamous cell carcinoma cell line (Tca8113)	Overexpression, knockdown	E-cadherin	Vimentin	Wnt/ β -catenin	[149]
	Breast cancer cell line (MDA-MB-231)	Knockdown	Vimentin, Slug	E-cadherin	N. D	[187]
	Breast cancer (MCF7), colon cancer (HCT116) and prostate cancer (DU145) cell lines	Exogenous added, blockade with lactose	E-cadherin	ZEB1	Trop2/β –catenin	[186]
	Breast cancer cell line (GI-LM2)	Knockdown	E-cadherin	Vimentin	Inhibition of Wnt and AKT pathways	[151]
**Gal-4**	Prostate cancer cell lines (PC-3, 22Rv1, LNCaP and DU-145) and mice	Knockdown, blockade with lactose	E-cadherin	Fibronectin, vimentin, Twist	ERK, AKT	[152]
**Gal-8**	MDCK cell line	Overexpression, blockade with lactose	E-cadherin	Vimentin, Snail, fibronectin, α5 Integrin, MMP-13	FAK/EGFR/ERK	[153]
	Primary prostate cancer cell line	Knockdown	E-cadherin	N. D	N. D	[189]

### Concluding remarks

Galectins have the potential to promote EMT in different contexts by modulating intracellular signaling pathways through their glycan-mediated interactions with plasma membrane receptors. In tissue repair, galectins can stimulate cell migration and re-epithelization with the consequential acceleration of wound healing after injury, as shown for Gal-2, Gal-3, Gal-4 and Gal-7 in different model systems. Evidence suggests that these galectins may induce tissue repair-associated EMT. In fibrosis, the evidence mainly derived by studies on Gal-1 and Gal-3 strongly suggests a pathogenic influence of overexpression of these galectins in a type of EMT considered to perpetuate a chronic stimulation of ECM depositing cells. The mechanisms include a mutual cooperation with known EMT-promoting factors, such as TGF-β1. In cancer, galectin overexpression has been found in different carcinomas and many studies combining experiments in a variety of cell lines and xenografts models, together with the analysis of human tissues and survival data, support a role of galectins promoting an EMT that enhances cell proliferation, invasive migration and metastasis, thus contributing to worsen the prognostics. Most studies refer to Gal-1, Gal-3, Gal-4, and Gal-8 involving different cell surface receptors and signaling pathways depending on the cell types. An exception is the protecting role of Gal-3 in breast cancer. Gal-8 provides a unique example of how a galectin overexpression can lead to pro-tumorigenic EMT in otherwise non-tumoral epithelial cells. The evidence involves Gal-8 in pro-invasive and metastatic EMT.

Many aspects regarding the mechanisms by which galectins promote EMT require further definition. Their interaction with extracellular components post-secretion is usually tested, but an intracellular role cannot be ruled out as this possibility is currently not experimentally evaluated. The signaling receptors and intracellular pathways and the EMT-TFs induced by galectins in different physiologic and pathogenic contexts, as well as the role of modifications of the sugar code, remain to be elucidated. Potential interrelations between different galectins, including whether one galectin influences the expression of other galectins and how they might synergistically contribute or counteract the induction of EMT needs to be clarified. A more comprehensive understanding of all these aspects is crucial for defining the potential therapeutic use of galectins or galectin inhibitors in processes of EMT associated with tissue repair, fibrosis and cancer.

## Data Availability

Not applicable.
